# Preoperative dermatomal somatosensory evoked potentials in risk prediction of early postoperative neurological deterioration after thoracic spine surgery: a retrospective cohort study

**DOI:** 10.1186/s13018-026-06992-0

**Published:** 2026-05-29

**Authors:** Yongjie Zhang, Yuan Liu, Lixuan Wang, Yuchen Wang, Jialiang Li, Huaguang Qi, Yang Yuan

**Affiliations:** 1 Department of Functional Examination, Xi’an Honghui Hospital, Xi’an Jiaotong University, Xi’an, China; 2 Disc and Deformity Ward, Hospital for Spinal Diseases, Xi’an Honghui Hospital, Xi’an Jiaotong University, Xi’an, China

**Keywords:** Thoracic spine, Spinal cord compression, Functional reserve, Dermatomal somatosensory evoked potentials, Risk prediction

## Abstract

**Background:**

Early postoperative neurological deterioration remains one of the most serious complications after thoracic spine surgery. Conventional preoperative risk assessment relies mainly on clinical and imaging variables, whereas the incremental value of electrophysiological indicators has not been fully defined. Dermatomal somatosensory evoked potentials (DSEP) may provide segment-specific functional information beyond structural imaging. This study aimed to develop and internally validate a preoperative prediction model incorporating electrophysiological parameters for early postoperative neurological deterioration after thoracic decompression surgery.

**Methods:**

A total of 508 patients who underwent thoracic decompression surgery were retrospectively included. Candidate predictors comprised age, preoperative Japanese Orthopaedic Association (JOA) score, number of compressed levels, T2-weighted intramedullary signal change, and DSEP-derived variables including maximal N1 latency, minimal amplitude, and number of abnormal DSEP sites. Four multivariable logistic regression models were constructed: a clinical model, a clinical + imaging model, a clinical + electrophysiological model, and a combined model. Internal validation was performed using stratified five-fold cross-validation. Model performance was assessed using the area under the receiver operating characteristic curve (AUC), calibration metrics, Brier score, decision curve analysis, and bootstrap-based optimism correction.

**Results:**

Among the 508 patients, 107 (21.1%) met criteria for early postoperative neurological deterioration. In the combined multivariable model, T2-weighted intramedullary signal change (OR 1.734, 95% CI 1.071–2.809, *P* = 0.025) and the number of abnormal DSEP sites (OR 1.687, 95% CI 1.338–2.128, *P* < 0.001) were independently associated with early postoperative neurological deterioration, whereas maximal N1 latency showed a borderline association (OR 1.092, 95% CI 0.997–1.196, *P* = 0.058). Cross-validated discrimination was limited for the clinical model (AUC 0.543, 95% CI 0.478–0.610) and improved modestly for the clinical + imaging model (AUC 0.596, 95% CI 0.536–0.659). Incorporation of electrophysiological variables substantially improved performance in the clinical + electrophysiological model (AUC 0.713, 95% CI 0.655–0.766), while the combined model showed the highest overall performance (AUC 0.715, 95% CI 0.658–0.770). Models containing electrophysiological variables also demonstrated better calibration and lower Brier scores. Bootstrap optimism correction showed only modest optimism across models.

**Conclusions:**

Preoperative DSEP abnormalities, particularly a greater burden of abnormal DSEP sites, may improve risk stratification for early postoperative neurological deterioration after thoracic spine surgery. Models incorporating electrophysiological variables showed better discrimination, calibration, and clinical net benefit than models based on clinical variables alone. These findings support the potential role of preoperative DSEP as a functional adjunct to conventional assessment, but external validation is required before broader clinical implementation.

**Supplementary Information:**

The online version contains supplementary material available at 10.1186/s13018-026-06992-0.

## Background

Thoracic spinal cord compression is a relatively uncommon spinal disorder, yet the uncertainty of neurological recovery following surgical treatment is significantly greater than that observed in cervical pathology [1]. Although decompressive surgery aims to relieve mechanical compression of the spinal cord and create conditions favorable for neurological recovery, clinical practice frequently reveals paradoxical findings: some patients with radiologically complete decompression experience limited neurological improvement, whereas others with similar degrees of preoperative compression demonstrate markedly different postoperative recovery trajectories [2, 3]. These observations strongly suggest that postoperative neurological outcomes are not determined solely by the extent of mechanical decompression and may involve more complex pathophysiological mechanisms [4].

Currently, preoperative assessment primarily depends on imaging findings and clinical functional scores. Conventional imaging modalities such as magnetic resonance imaging (MRI) mainly depict morphological structural alterations rather than functional integrity of neural conduction pathways [5, 6]. T2-weighted intramedullary hyperintensity is often interpreted as a marker of spinal cord edema, gliosis, or cystic degeneration; however, its association with prognosis remains controversial [7]. Multiple studies have demonstrated that the relationship between T2 signal change and neurological recovery is inconsistent. Some patients with pronounced signal alterations achieve favorable postoperative recovery, whereas others with minimal imaging abnormalities show poor outcomes. This “imaging–clinical paradox” highlights the limitation of purely morphological assessment, as imaging findings represent static structural alterations and fail to reflect the dynamic functional state of axonal conduction within the spinal cord [8].

Neurophysiological assessment provides an important functional complement to imaging evaluation. In the pathological process of spinal cord compression, abnormalities in neural conduction frequently precede irreversible structural damage. Somatosensory evoked potentials (SEP), which assess dorsal column (gracile and cuneate fasciculi) sensory conduction, theoretically offer early warning value [9–11]. However, conventional mixed-nerve SSEP recordings represent composite potentials conducted through long ascending pathways to the cortex, and waveform characteristics and latency are influenced by multiple individual factors. These confounders include height-related physiological variation, peripheral neuropathy secondary to metabolic disorders, and coexisting lumbar or cervical stenosis, all of which may independently alter SSEP results [3, 12, 13]. Even under intraoperative monitoring conditions using self-controlled comparisons, interpretation may be influenced by anesthetic regimen, core temperature, mean arterial pressure, and surgical manipulation [11]. Therefore, although SEP is widely applied in intraoperative monitoring, its sensitivity and specificity as an independent preoperative predictor of postoperative functional recovery remain limited.

Given the limitations of traditional imaging and electrophysiological indicators in predicting prognosis of thoracic spinal cord compression, this study proposed integration of dermatomal somatosensory evoked potentials (DSEP) with clinical variables to construct a multidimensional prediction model [9, 14]. Compared with SSEP, DSEP records conduction from specific dermatomes or peripheral nerves, allowing more precise localization and evaluation of segmental nerve root and spinal cord function, and potentially more sensitively reflecting the impact of localized compression on neural conduction pathways.

The objectives of this study were:

1. To systematically evaluate the association between preoperative DSEP characteristics and postoperative neurological outcomes;
2. To identify independent risk factors through multivariable analysis incorporating clinical variables;
3. To develop and internally validate a prognostic prediction model and assess its clinical applicability for preoperative risk stratification.

We hypothesized that DSEP-derived functional conduction parameters would compensate for the inability of imaging to evaluate functional impairment, thereby more accurately identifying patients most likely to benefit from surgical intervention.

## Methods

### Study design and population

This was a single-center retrospective cohort study. Consecutive patients who underwent thoracic decompression surgery at Xi’an Honghui Hospital, Xi’an Jiaotong University, were screened for eligibility. Inclusion criteria were: (1) radiologically confirmed thoracic spinal cord compression; (2) standard posterior or combined decompression surgery; (3) completion of preoperative DSEP examination; and (4) availability of complete perioperative follow-up data. Exclusion criteria were: (1) history of prior spinal cord injury; (2) severe peripheral neuropathy or major metabolic neurological disorders; and (3) incomplete data or loss to follow-up. All patients had provided written informed consent for electrophysiological examination. Because preoperative DSEP examination requires specialized equipment, technical expertise, and relatively long acquisition time, it was clinically performed mainly in patients with more severe neurological involvement, complex thoracic cord compression, or concern regarding spinal cord functional reserve. Therefore, the present cohort should be interpreted as a selected high-risk surgical population rather than an unselected thoracic spine surgery population. The study was approved by the institutional ethics committee and conducted in accordance with the Declaration of Helsinki.

### Preoperative DSEP examination

All electrophysiological examinations were performed preoperatively by the same neurophysiological team to ensure methodological consistency. Testing was conducted in a sound-attenuated room with ambient temperature maintained at 24–26 °C. Patients were positioned supine comfortably. Surface stimulation electrodes were placed bilaterally along the midaxillary line corresponding to thoracic dermatomes: T2 at the sternal angle level, T4 at the nipple level, T6 at the xiphoid level, T8 at the costal arch level, T10 at the umbilical level, and T12 at the midpoint between the umbilicus and pubic symphysis. Stimulation intensity was set at 2–3 times the sensory threshold, with a stimulation frequency of 3 Hz.

Signals were recorded using a Nihon Kohden electromyography system. Subdermal monopolar needle electrodes were positioned according to the international 10–20 system, with the active recording electrode at Cz′ (2 cm posterior to Cz) and the reference electrode at Fz. Each stimulation site was averaged over 100 trials and repeated twice to ensure waveform stability and reproducibility.

Measured parameters included N1 latency, P1 latency, peak-to-peak amplitude, and the number of abnormal DSEP sites.

### Definition of abnormal DSEP findings

For each dermatomal recording site, N1 latency and peak-to-peak amplitude were evaluated. The reference standards are derived from the data previously published by our research team [15]. Segment-specific normal reference values for N1 latency were 15.04 ± 1.74 ms at T2, 17.27 ± 2.13 ms at T4, 18.07 ± 1.83 ms at T6, 18.50 ± 1.70 ms at T8, 20.13 ± 1.55 ms at T10, and 21.52 ± 2.12 ms at T12. Prolonged latency was defined as an N1 latency exceeding the upper limit of normal, calculated as the segment-specific mean plus 2 standard deviations, corresponding to thresholds of > 18.52 ms at T2, > 21.53 ms at T4, > 21.73 ms at T6, > 21.90 ms at T8, > 23.23 ms at T10, and > 25.76 ms at T12. Amplitude abnormality was defined as a reduction of at least 50% relative to the contralateral side or waveform absence. In cases of waveform absence, the recording was classified as abnormal regardless of contralateral amplitude. Bilateral recordings were evaluated separately; therefore, bilateral abnormalities at the same dermatomal level were counted as two abnormal DSEP sites. Maximal N1 latency was defined as the highest N1 latency observed across all evaluable bilateral dermatomal recordings, and minimal amplitude was defined as the lowest peak-to-peak amplitude across all evaluable recordings.

### Clinical and imaging data collection

Collected variables included age, preoperative Japanese Orthopaedic Association (JOA) score, number of compressed levels, operative time, and the presence of T2-weighted intramedullary signal change on MRI. Neurological function was assessed using the Japanese Orthopaedic Association (JOA) score (range 0–11), with lower scores indicating more severe impairment. MRI findings were independently evaluated by two spine surgeons, and any discrepancies were resolved by consensus.

### Outcome definition

The primary outcome was early postoperative neurological deterioration within 3 months after surgery. This outcome was defined as new-onset neurological deficit or clinically meaningful worsening compared with the preoperative neurological status, supported by neurological examination and postoperative functional assessment using the JOA score. Neurological deterioration included new or aggravated motor weakness, sensory decline, or deterioration in functional neurological status documented during postoperative follow-up. Event classification was based on documented neurological findings together with postoperative functional assessment, rather than subjective symptoms alone. Because the outcome was assessed within the early postoperative period, it should be interpreted as early neurological deterioration rather than permanent neurological injury.

### Candidate predictors and variable selection strategy

Candidate predictors were prespecified according to clinical relevance and availability before model fitting. Clinical variables included age, preoperative JOA score, and number of compressed levels. Imaging was represented by the presence or absence of T2-weighted intramedullary signal change. Electrophysiological predictors included maximal N1 latency, minimal amplitude, and the number of abnormal DSEP sites. Operative time was considered a candidate perioperative variable but was not included in the primary preoperative prediction models because it is not available at the time of preoperative risk estimation and may overlap conceptually with surgical complexity. Accordingly, operative time was evaluated in sensitivity analyses rather than in the primary models.

### Model development and internal validation

Four multivariable logistic regression models were constructed: (1) a clinical model; (2) a clinical + imaging model; (3) a clinical + electrophysiological model; and (4) a combined model including clinical, imaging, and electrophysiological variables.

Internal validation was primarily performed using stratified five-fold cross-validation with shuffled splits. In each fold, the model was fitted in the training subset and applied to the held-out subset to generate out-of-fold predicted probabilities, which were then aggregated for performance evaluation.

### Assessment of multicollinearity

Potential collinearity among candidate predictors was assessed before multivariable modeling using pairwise correlations and variance inflation factors (VIFs). Variables considered to reflect overlapping clinical constructs, particularly operative time and number of compressed levels, were reviewed for conceptual redundancy. Because operative time reflects intraoperative complexity rather than purely preoperative risk, it was reserved for sensitivity analyses. No substantial multicollinearity was observed among predictors included in the primary combined model.

### Performance assessment and sensitivity analyses

Model discrimination was assessed using the area under the receiver operating characteristic curve (AUC). Calibration was evaluated using quantile-based calibration curves with ten equal-frequency bins, as well as calibration intercept, calibration slope, and Brier score. Decision curve analysis was performed across threshold probabilities ranging from 0.01 to 0.50.

To further assess internal optimism, bootstrap resampling was used to estimate apparent AUC, optimism-corrected AUC, and optimism values for each model. Sensitivity analyses were also performed by adding operative time to the combined model and by refitting the combined model after excluding selected predictors.

### Statistical analysis

Continuous variables were summarized as median [interquartile range] and compared using appropriate nonparametric tests. Categorical variables were summarized as number (percentage) and compared using chi-square or Fisher’s exact tests, as appropriate. All statistical analyses were performed using Python (version 3.12). Full regression coefficients, including intercepts, are provided in the Supplementary Material to facilitate independent validation and application.

## Results

### Baseline characteristics and univariable comparisons

A total of 508 patients undergoing thoracic decompression surgery were included, of whom 107 (21.1%) met the predefined criteria for early postoperative neurological deterioration within 3 months. Age and preoperative JOA score did not differ significantly between groups. In contrast, patients with early postoperative neurological deterioration had significantly longer operative time, a greater number of compressed levels, higher maximal N1 latency, and a greater number of abnormal DSEP sites. T2-weighted intramedullary signal change and bilateral DSEP abnormality were also more frequent in the deficit group. Detailed baseline comparisons are presented in Table 1.

**Table 1:** Baseline characteristics according to neurological outcome

Variable	No deterioration (*n* = 401)	Deterioration (*n* = 107)	*P* value
Age (years)	59.00 [50.00, 67.00]	59.00 [52.50, 65.50]	0.838
JOA preop	6.60 [4.90, 7.90]	6.40 [4.40, 8.80]	0.736
Male sex	197 (49.1%)	47 (43.9%)	0.396
*Compressed levels, n*			0.007
1	298 (74.3%)	67 (62.6%)	
2	82 (20.4%)	25 (23.4%)	
3	21 (5.2%)	15 (14.0%)	
Operative time (min)	196.00 [174.00, 219.00]	203.00 [188.00, 225.00]	0.005
DSEP max N1 latency (ms)	20.79 [18.54, 22.81]	22.86 [20.64, 24.47]	< 0.001
DSEP min amplitude	0.28 [0.16, 0.38]	0.25 [0.16, 0.36]	0.307
Abnormal DSEP sites, n	1.00 [1.00, 2.00]	3.00 [2.00, 3.00]	< 0.001
T2 signal change	106 (26.4%)	49 (45.8%)	< 0.001
Any absent DSEP	66 (16.5%)	14 (13.1%)	0.483
Bilateral DSEP abnormality	105 (26.2%)	60 (56.1%)	< 0.001

Values are presented as median [interquartile range] or n (%)

### Comparative performance of prediction models

**Fig. 1:**
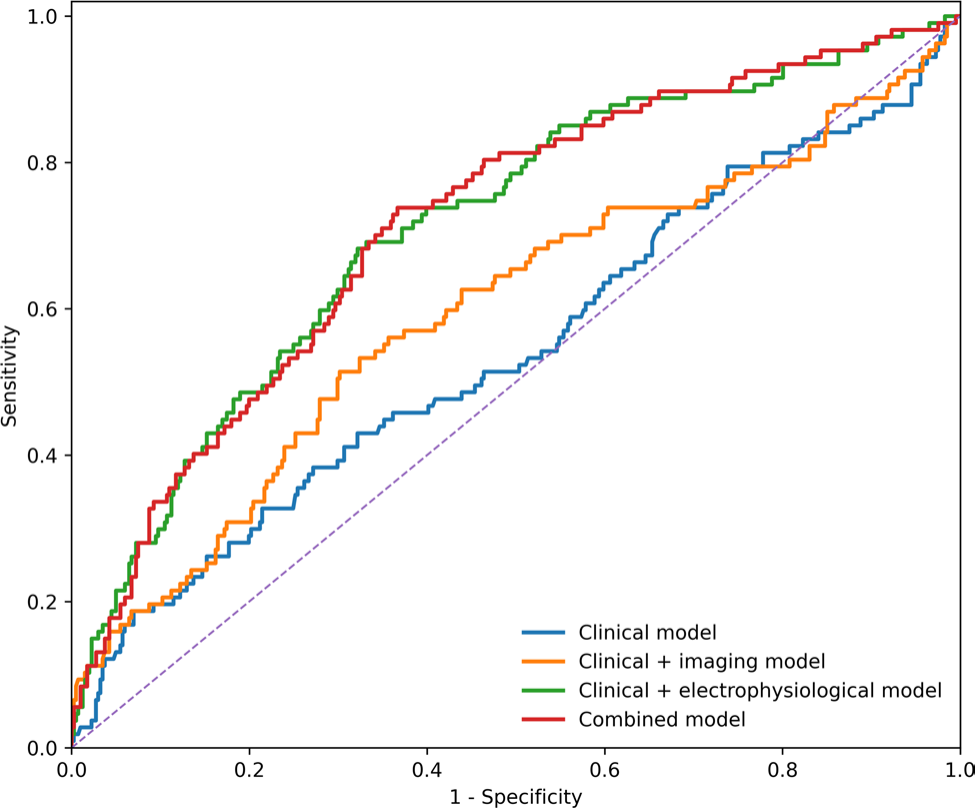
Receiver operating characteristic (ROC) curves comparing the predictive performance of four models for early postoperative neurological deterioration

Internal validation using stratified five-fold cross-validation showed limited discrimination for the clinical model (AUC = 0.543, 95% CI 0.478–0.610). Addition of MRI signal change yielded modest improvement in the clinical + imaging model (AUC = 0.596, 95% CI 0.536–0.659). Incorporation of electrophysiological variables substantially improved discrimination, with the clinical + electrophysiological model achieving an AUC of 0.713 (95% CI 0.655–0.766). The combined model showed the highest overall performance (AUC = 0.715, 95% CI 0.658–0.770). Detailed model performance metrics are presented in Table 2, and ROC curves are shown in Fig. 1.

**Table 2:** Internal validation performance of the four prediction models

Model	Cross-validated AUC	95% CI	Brier score	Calibration intercept	Calibration slope
Clinical model	0.543	0.478–0.610	0.165	− 0.014	0.670
Clinical + imaging model	0.596	0.536–0.659	0.161	− 0.019	0.821
Clinical + electrophysiological model	0.713	0.655–0.766	0.149	− 0.021	0.960
Combined model	0.715	0.658–0.770	0.149	− 0.018	0.935

AUC, area under the receiver operating characteristic curve; CI, confidence interval

### Calibration, internal optimism, and clinical utility

**Fig. 2:**
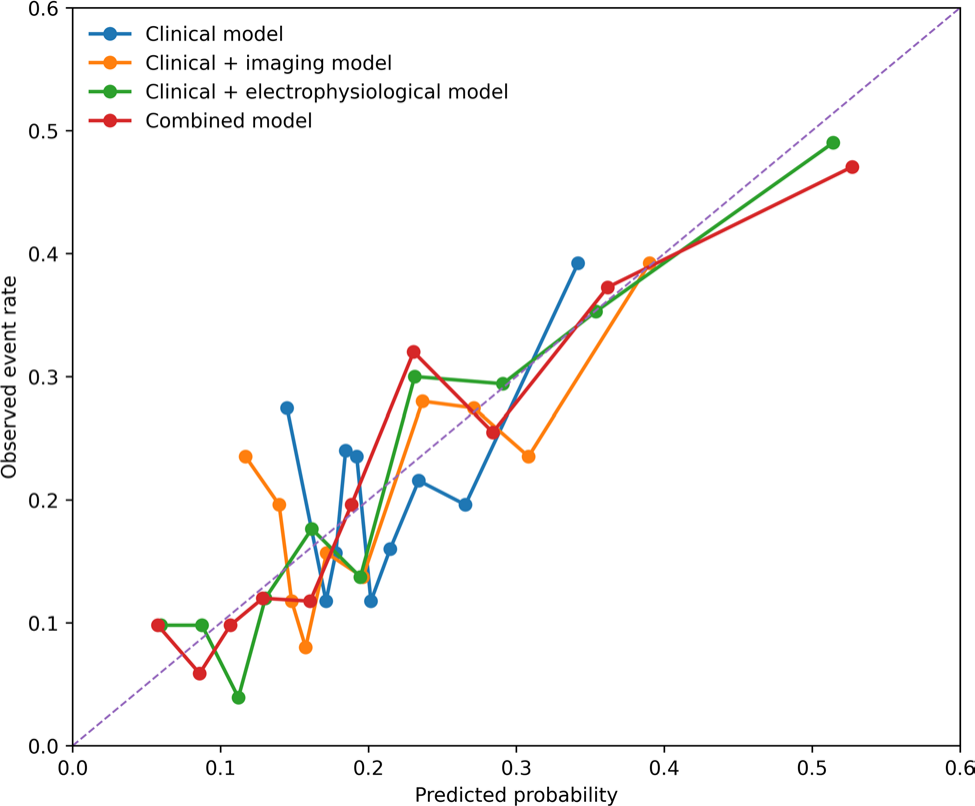
Calibration curves of the four predictive models using ten equal-frequency bins. The diagonal dashed line indicates perfect calibration

Calibration analysis showed that models incorporating electrophysiological variables had more favorable calibration performance than the clinical-only and clinical + imaging models. The calibration slope was 0.960 for the clinical + electrophysiological model and 0.935 for the combined model, compared with 0.670 for the clinical model. Brier scores were also lower for the electrophysiology-containing models (both 0.149) than for the clinical-only model (0.165). Calibration plots are shown in Fig. 2.

Bootstrap-based optimism correction demonstrated only modest optimism across models. Apparent and optimism-corrected AUC values are summarized in Supplementary Table S3, and the relationship between apparent and optimism-corrected AUC values across the four models is illustrated in Supplementary Figure S1. The distribution of optimism values across bootstrap resamples is shown in Supplementary Figure S2. The corresponding optimism values were small, supporting reasonable internal stability of the developed models.

**Fig. 3:**
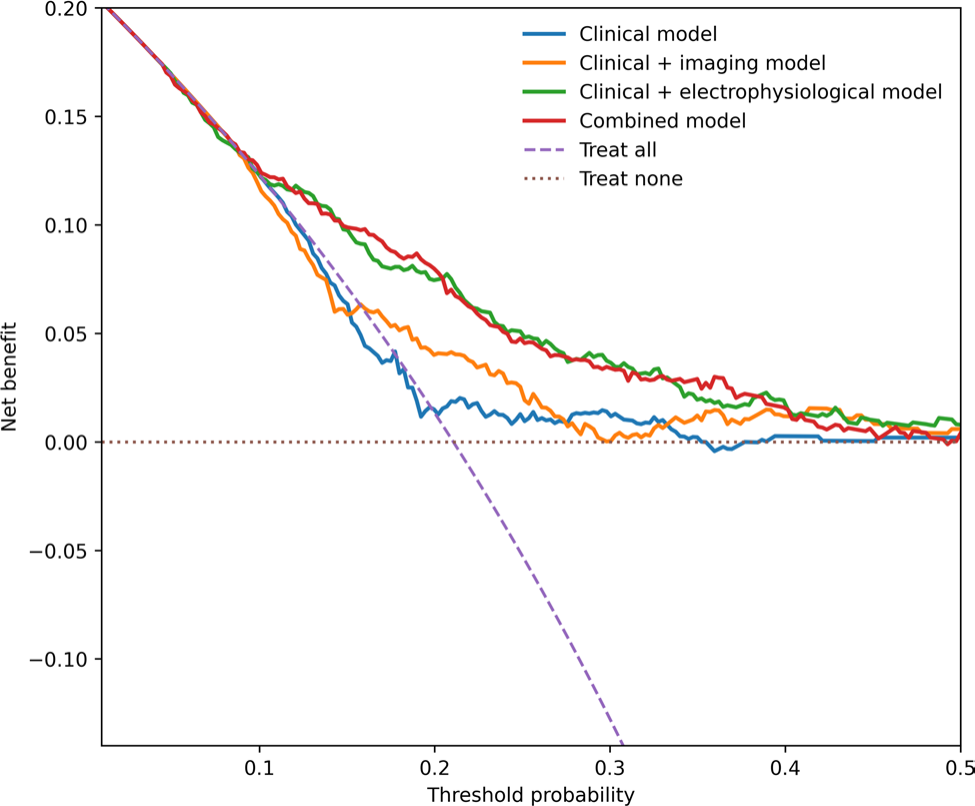
Decision curve analysis comparing the net clinical benefit of different models across threshold probabilities from 0.01 to 0.50. Treat-all and treat-none strategies are shown for reference

Decision curve analysis demonstrated greater net benefit for the clinical + electrophysiological and combined models across clinically relevant intermediate threshold probabilities, particularly in the range of approximately 0.15 to 0.30. These findings suggest that the electrophysiology-containing models may be most useful when the predicted risk is used to identify patients warranting intensified perioperative monitoring and counseling (Fig. 3).

### Multivariable logistic regression analysis

Formal assessment of multicollinearity showed no substantial collinearity among predictors included in the primary combined model, with all variance inflation factors below 2 (Supplementary Table S1).

In the combined multivariable logistic regression model, T2-weighted intramedullary signal change (OR 1.734, 95% CI 1.071–2.809, *P* = 0.025) and the number of abnormal DSEP sites (OR 1.687, 95% CI 1.338–2.128, *P* < 0.001) were independently associated with early postoperative neurological deterioration. Maximal N1 latency showed a borderline association with outcome (OR 1.092, 95% CI 0.997–1.196, *P* = 0.058). Age, preoperative JOA score, number of compressed levels, and minimal amplitude were not independently associated with outcome in the final combined model.

The regression results for the final combined model are shown in Table 3, whereas full regression coefficients for all four models, including intercepts, are provided in Supplementary Table S2B.

**Table 3:** Multivariable logistic regression results for the final combined model

Predictor	β coefficient	SE	OR	95% CI	*P* value
Age	− 0.001	0.010	0.999	0.980–1.018	0.928
Preoperative JOA score	0.035	0.049	1.036	0.941–1.141	0.473
Number of compressed levels	0.309	0.179	1.362	0.960–1.934	0.084
T2-weighted intramedullary signal change	0.551	0.246	1.734	1.071–2.809	0.025
Maximal N1 latency	0.088	0.046	1.092	0.997–1.196	0.058
Minimal amplitude	− 0.363	0.919	0.695	0.115–4.211	0.693
Number of abnormal DSEP sites*	0.523	0.118	1.687	1.338–2.128	< 0.001

JOA, Japanese Orthopaedic Association; DSEP, dermatomal somatosensory evoked potential; OR, odds ratio; CI, confidence interval; SE, standard error. Intercept = − 5.029. *Although previously referred to as the “number of abnormal segments,” this variable was calculated on a side-specific basis

### Sensitivity analyses

Sensitivity analyses yielded findings broadly consistent with the primary model. Adding operative time to the combined model slightly increased discrimination but did not materially alter the direction or relative importance of the main imaging and electrophysiological predictors. Similarly, exclusion of minimal amplitude or T2-weighted signal change produced only limited changes in overall performance, supporting the robustness of the primary findings. Detailed sensitivity analysis results are provided in Supplementary Table S2A.

## Discussion

In this cohort of patients undergoing thoracic decompression surgery, adding preoperative DSEP to conventional assessment improved postoperative risk prediction. This improvement was not limited to ROC discrimination. Models containing electrophysiological variables also showed better calibration, lower Brier scores, and only modest optimism after bootstrap correction. Taken together, these findings suggest that DSEP is capturing clinically relevant functional information that is not fully reflected by routine clinical variables or by a simple structural imaging marker alone.

The observed event rate in this cohort was 21.1%, which is higher than the previously reported rate of 13.9% for neurological deficits after thoracic decompression [16]. Nevertheless, the literature supports that thoracic decompression is associated with a relatively high risk of neurological complications. This higher event rate should be interpreted in the context of both patient selection and outcome definition. First, preoperative DSEP was not performed as a routine screening test for all thoracic spine surgery patients in our center; rather, it was generally used in patients with more severe neurological involvement, complex thoracic cord compression, or greater concern regarding spinal cord functional reserve. Therefore, the present cohort represents a selected high-risk surgical population rather than an unselected thoracic spine surgery population. This interpretation is also supported by the baseline profile of the cohort, in which a substantial proportion of patients showed multilevel compression, T2-weighted intramedullary signal change, and abnormal DSEP findings. Second, the outcome in this study reflected early neurological deterioration within 3 months after surgery, rather than permanent neurological injury. Transient or partially reversible neurological worsening may therefore have been included. Accordingly, the reported event rate should not be directly compared with studies focusing only on permanent or severe postoperative neurological complications.

What stands out most in the present results is that the overall burden of thoracic electrophysiological abnormality seemed to matter more than any single latency value by itself. In the final combined model, the number of abnormal DSEP sites remained independently associated with early postoperative neurological deterioration, whereas maximal N1 latency showed only a borderline association [6, 9]. Although the number of abnormal DSEP sites may partially overlap with the number of compressed levels, the two variables represent different constructs. The number of compressed levels reflects structural disease extent on imaging, whereas the number of abnormal DSEP sites represents side-specific functional conduction abnormality. Bilateral abnormalities at the same dermatomal level were counted separately, meaning that this variable captured functional burden rather than simply the anatomical number of compressed segments. The absence of substantial multicollinearity supports, but does not prove, that these variables contributed distinct information. Therefore, the number of abnormal DSEP sites should be interpreted as a predictive marker of functional burden rather than a purely independent mechanistic factor. This pattern is clinically plausible. In thoracic compressive myelopathy, outcome is unlikely to be determined by one delayed response at one level alone. More often, it probably depends on how much of the thoracic cord has already lost segment-specific functional reserve. Our data do not prove that directly, but they do suggest that broader dermatomal dysfunction carries more prognostic information than an isolated latency abnormality [3].

This also fits with the known neurophysiology of chronic cord compression. N1 latency reflects conduction along the dorsal column pathway, and prolonged latency usually indicates slowed neural transmission related to chronic stress on the cord, including myelin injury, nodal dysfunction, or longstanding compression [17–19]. But from a clinical point of view, a patient with multiple thoracic dermatomes showing abnormal conduction is usually more concerning than a patient with one mildly prolonged waveform. In that sense, DSEP may be useful not simply because it detects abnormality, but because it gives a more segment-specific picture of how much function remains across the thoracic cord.

That idea becomes more understandable if DSEP is viewed as a marker of preoperative functional vulnerability. In chronic compressive myelopathy, the spinal cord is exposed to more than just static mechanical narrowing. Persistent compression may be accompanied by impaired conduction, reduced microcirculatory reserve, and greater susceptibility to secondary injury during and after decompression [9, 20–22]. DSEP abnormalities may therefore reflect a cord that is already physiologically stressed and less able to tolerate further perioperative insult. We did not directly measure blood–spinal cord barrier dysfunction, ischemia–reperfusion injury, or other mechanistic processes in this study, so the biological interpretation should remain cautious. Still, the overall pattern of our results is consistent with the idea that DSEP provides a functional signal that conventional structural assessment alone cannot fully provide [17, 22, 23].

**Fig. 4:**
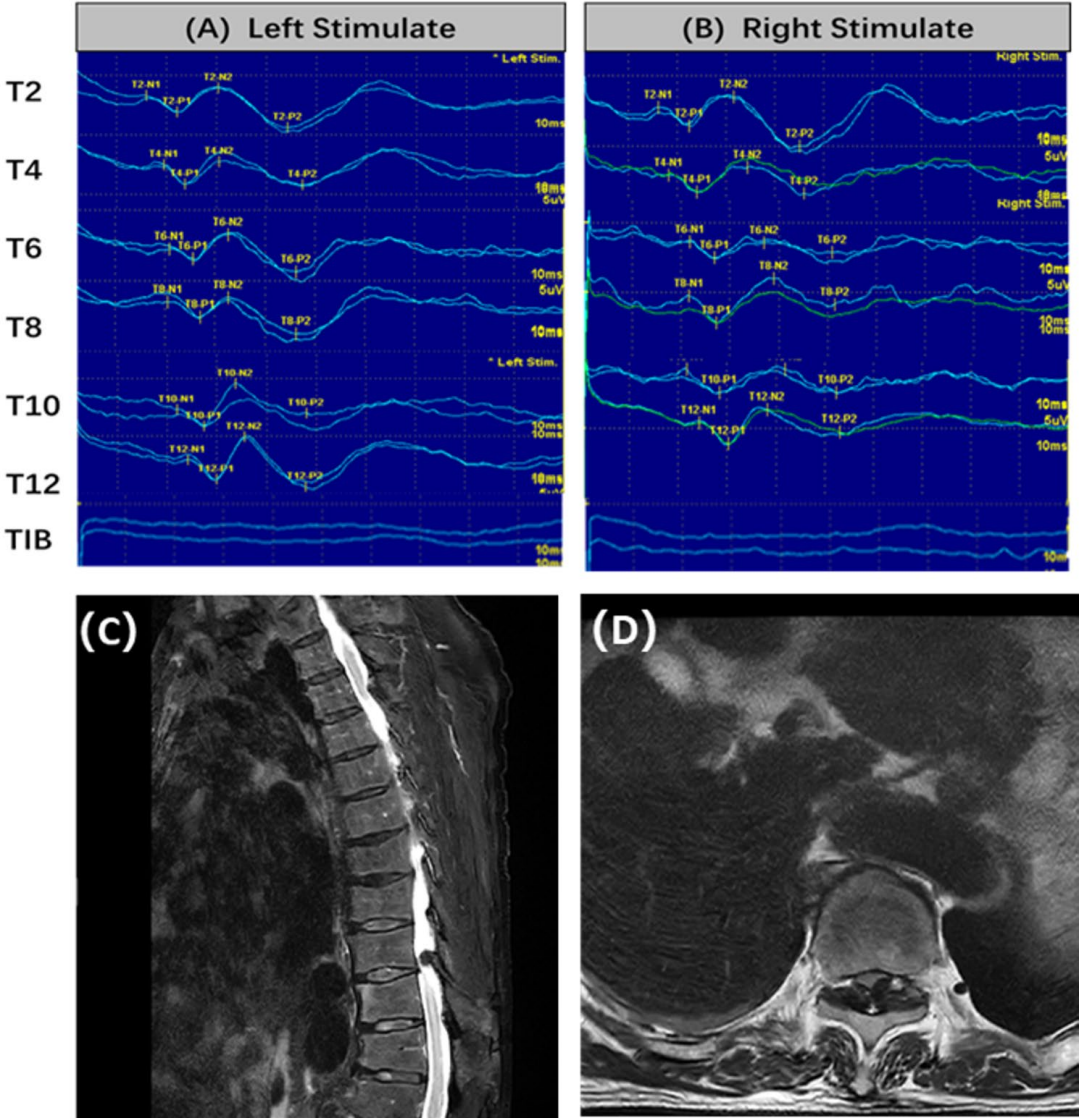
**A** and **B** Preoperative electrophysiological recordings obtained during left- and right-sided stimulation in an 81-year-old male patient. Thoracic DSEP waveforms were identifiable from T2 to T12, whereas no reproducible CSEP waveform was observed after medial malleolus stimulation. **C** and **D** Sagittal and axial T2-weighted MRI of the thoracic spine, showing spinal cord compression with intramedullary T2 hyperintensity. postoperative neurological improvement was confirmed clinically

One representative case from our cohort illustrates this point clearly (Fig. 4). An 81-year-old male patient presented with severe thoracic spinal canal stenosis. Preoperatively, he had obvious neurological dysfunction, with lower limb muscle strength at approximately grade 3. He also had sensory deficits in both lower limbs, and his overall neurological function was poor. The preoperative JOA score was 4. As shown in Fig. 4C and D, sagittal and axial T2-weighted magnetic resonance imaging revealed significant compression of the thoracic spinal cord, accompanied by intramedullary high signal changes, indicating possible spinal cord injury. The results of the conventional lower limb CSEP were significantly abnormal, with both sides showing absent responses. In contrast, the thoracic DSEP recordings remained good. As shown in Fig. 4A and B, despite severe structural compression, clear thoracic DSEP waveforms could still be identified from T2 to T12, indicating that the segmental sensory conduction within the thoracic spinal cord remained at a meaningful level. This difference is clinically significant. The patient also suffered from severe lumbar degenerative disease, which may have led to abnormal CSEP test results and reduced the specificity of his thoracic functional reserve. After undergoing thoracic decompression surgery, his neurological condition improved, with lower limb muscle strength returning to approximately grade 4, and some sensory symptoms improved; accordingly, his postoperative JOA score was estimated to have increased to 7. This case highlights a common pattern in spinal treatment: the results of conventional imaging examinations and lower limb electrophysiological tests may seem discouraging, but postoperative recovery may still be better than expected. In this situation, thoracic DSEP may provide a more anatomically relevant indicator to reflect the preservation of thoracic segmental function.

The MRI findings in this study should be interpreted in the same spirit. In the final combined model, T2-weighted intramedullary signal change remained independently associated with early postoperative neurological deterioration, but the contribution of imaging to overall model improvement was modest compared with the electrophysiological variables. We do not think this means MRI is of limited value. More likely, it reflects the fact that a binary T2 signal variable is too crude to represent the full biological spectrum of cord injury [6, 24, 25]. T2 hyperintensity may reflect edema, inflammation, ischemic change, gliosis, or myelomalacia, and these do not carry the same prognostic meaning. In a cohort already selected for surgery because of significant symptoms, such a simplified imaging variable may have only limited discriminatory value once functional measures are entered into the same model [26]. This is also why more advanced quantitative MRI techniques, such as diffusion tensor imaging, magnetization transfer imaging, or myelin-sensitive imaging, remain important directions for future work [27–29].

Age is another result that needs a careful reading. Older age is clinically plausible as a risk marker, but it did not remain independently associated with outcome in the final combined model. One explanation is that part of the risk signal carried by age is already being captured by imaging and electrophysiological variables. Another is that age may be acting as a proxy for other unmeasured factors [30, 31]. Older patients are more likely to have hypertension, diabetes, cardiovascular disease, and other vascular or metabolic conditions that may affect spinal cord perfusion and recovery potential. Because these factors were not systematically collected in the present study, some residual confounding is unavoidable. For that reason, the association between DSEP abnormalities and postoperative risk should not be interpreted as completely independent of broader patient vulnerability [32, 33].

From a modeling perspective, the contribution of DSEP in this study is meaningful, but it should be described in proportion. The improvement in AUC was real and consistent, and it was supported by calibration, Brier score, optimism correction, and sensitivity analyses. The final combined model included seven predictors and 107 outcome events, corresponding to an events-per-variable ratio of approximately 15. This ratio is generally considered acceptable for logistic regression modeling and, together with stratified cross-validation and bootstrap optimism correction, supports reasonable internal stability. However, this does not eliminate the need for external validation. At the same time, the final model is not a perfect predictor, and the overall performance remains moderate rather than definitive. This is important when positioning the study in the literature. The present work should be seen as an incremental advance, not a transformative one. Its value lies in showing, in a thoracic surgery cohort, that segment-specific preoperative electrophysiological assessment adds useful information beyond routine evaluation and improves internal risk prediction in a clinically interpretable way. That may not resolve all of the uncertainties around postoperative recovery, but it is still a worthwhile step forward.

The clinical role of the model should also be framed realistically. It is not a tool for deciding whether surgery should or should not be performed. Rather, it is best viewed as a perioperative risk aid. In patients whose predicted risk falls within an intermediate range, especially where the decision curve showed the most meaningful net benefit, the model may help identify those who warrant closer intraoperative neuromonitoring attention, stricter hemodynamic management, more explicit counseling about neurological risk, and earlier postoperative rehabilitation planning. In that sense, the value of DSEP is not that it replaces routine judgment, but that it helps refine it [19, 21].

Several limitations should be kept in mind. First, because this was a retrospective single-center study based on patients who underwent clinically indicated preoperative DSEP examination, the cohort may represent a selected high-risk surgical population, and the observed event rate may not be generalizable to all patients undergoing thoracic spine surgery. Second, the outcome reflected early postoperative neurological deterioration within 3 months rather than permanent neurological injury; therefore, transient or reversible deficits may have been included. Longer-term follow-up is needed to determine whether preoperative DSEP abnormalities predict persistent neurological deficits or long-term functional recovery. Third, important comorbidities and chronicity-related variables, including diabetes, vascular disease, symptom duration, and baseline neurological chronicity, were not consistently available in this retrospective dataset, and residual confounding cannot be excluded. Only sensory pathway function was analyzed; motor pathway measures such as MEP may provide additional prognostic information. Imaging variables were limited to T2-weighted signal change, and more advanced quantitative MRI measures were not available. Accordingly, the association between DSEP abnormalities and early postoperative neurological deterioration should be interpreted as predictive rather than causal. Finally, DSEP testing requires specific equipment, technical expertise, and standardized acquisition procedures, which may limit wider implementation in some centers.

Overall, our findings suggest that preoperative DSEP can improve risk stratification for early postoperative neurological deterioration after thoracic decompression surgery by adding functional information that routine structural assessment does not fully provide. The results are encouraging, but they remain preliminary. Broader validation, more refined imaging integration, and further work on how best to apply DSEP in real perioperative decision-making will be needed before this approach can be used more confidently across centers.

## Conclusion

In this retrospective cohort of 508 patients undergoing thoracic decompression surgery, preoperative DSEP-based functional assessment improved preoperative risk stratification for early postoperative neurological deterioration. A greater number of abnormal DSEP sites and the presence of T2-weighted intramedullary signal change were independently associated with early postoperative neurological deterioration in the final combined model, while models incorporating electrophysiological variables demonstrated better discrimination, calibration, and clinical net benefit than models based on clinical variables alone. These findings suggest that preoperative DSEP may capture functional impairment not fully represented by conventional imaging variables and may serve as a useful adjunct in perioperative risk assessment. However, the results should be interpreted cautiously, as the study does not establish mechanism or broader clinical generalizability, and external validation is required before routine implementation.

## Supplementary Information

Below is the link to the electronic supplementary material.

Supplementary Material 1

## Data Availability

The datasets used and/or analysed during the current study are available from the corresponding author on reasonable request.
