# Supplementary material for: Preoperative dermatomal somatosensory evoked potentials in risk prediction of early postoperative neurological deterioration after thoracic spine surgery: a retrospective cohort study
Source: J Orthop Surg Res. 2026 May 29;21:459. doi: 10.1186/s13018-026-06992-0 (PMC13430841; doi:10.1186/s13018-026-06992-0)
Supplement: Supplementary file 1 — Supplementary Material 1 [file 13018_2026_6992_MOESM1_ESM.docx]

**Supplementary Materials**

**Supplementary Table S1. Variance inflation factor analysis for predictors included in the combined model**

| **Predictor** | **VIF** |
| --- | --- |
| Age | 1.011 |
| Preoperative JOA score | 1.011 |
| Number of compressed levels | 1.057 |
| T2-weighted intramedullary signal change | 1.067 |
| Maximal N1 latency | 1.363 |
| Minimal amplitude | 1.014 |
| Number of abnormal DSEP sites | 1.428 |

Interpretation: No substantial multicollinearity was observed among predictors included in the primary model, with all VIF values < 2.

**Supplementary Table S2A. Sensitivity analyses of the combined model**

| **Analysis** | **Cross-validated AUC** | **Brier score** | **Key findings** |
| --- | --- | --- | --- |
| Primary combined model | 0.715 | 0.149 | T2 signal change and abnormal DSEP sites remained significant; maximal N1 latency was borderline significant. |
| Combined model + operative time | 0.719 | 0.148 | Operative time was associated with increased risk; T2 signal change and abnormal DSEP sites remained significant. |
| Combined model without minimal amplitude | 0.718 | 0.149 | Model performance remained similar; abnormal DSEP sites remained significant. |
| Combined model without T2 signal change | 0.713 | 0.149 | Model discrimination changed minimally; abnormal DSEP sites remained significant. |

**Supplementary Table S2B. Full regression coefficients, including intercepts, for the four logistic regression models**

| **Panel A. Clinical and clinical + imaging models** | | |
| --- | --- | --- |
| **Term** | **Clinical model** | **Clinical + imaging model** |
| Intercept | -2.090 | -2.582 |
| Age | -0.004 | -0.001 |
|  |  |  |
| Preoperative JOA score | 0.040 | 0.039 |
| Number of compressed levels | 0.508 | 0.533 |
| T2-weighted intramedullary signal change | — | 0.876 |
| Maximal N1 latency | — | — |
| Minimal amplitude | — | — |
| Number of abnormal DSEP sites | — | — |
|  | | |
| **Panel B. Clinical + electrophysiological and combined models** | | |
| **Term** | **Clinical + electrophysiological model** | **Combined model** |
| Intercept | -4.887 | -5.029 |
| Age | -0.003 | -0.001 |
| Preoperative JOA score | 0.038 | 0.035 |
| Number of compressed levels | 0.277 | 0.309 |
| T2-weighted intramedullary signal change | — | 0.551 |
| Maximal N1 latency | 0.091 | 0.088 |
| Minimal amplitude | -0.225 | -0.363 |
| Number of abnormal DSEP sites | 0.570 | 0.523 |

Predicted probability equations:

For all models, P(postoperative neurological deficit) = 1 / (1 + exp(−Z)).

Clinical model: Z = −2.090 − 0.004×(Age) + 0.040×(Preoperative JOA score) + 0.508×(Number of compressed levels).

Clinical + imaging model: Z = −2.582 − 0.001×(Age) + 0.039×(Preoperative JOA score) + 0.533×(Number of compressed levels) + 0.876×(T2-weighted intramedullary signal change).

Clinical + electrophysiological model: Z = −4.887 − 0.003×(Age) + 0.038×(Preoperative JOA score) + 0.277×(Number of compressed levels) + 0.091×(Maximal N1 latency) − 0.225×(Minimal amplitude) + 0.570×(Number of abnormal DSEP sites).

Combined model: Z = −5.029 − 0.001×(Age) + 0.035×(Preoperative JOA score) + 0.309×(Number of compressed levels) + 0.551×(T2-weighted intramedullary signal change) + 0.088×(Maximal N1 latency) − 0.363×(Minimal amplitude) + 0.523×(Number of abnormal DSEP sites).

Variable coding: T2-weighted intramedullary signal change was coded as 0 = absent and 1 = present. All other predictors were entered as continuous variables.

**Supplementary Table S3. Apparent and optimism-corrected performance of the four prediction models**

| **Model** | **Apparent AUC** | **Optimism-corrected AUC** | **Optimism value** | **Bootstrap iterations used** | **Optimism 95% interval (bootstrap)** |
| --- | --- | --- | --- | --- | --- |
| Clinical model | 0.574 | 0.551 | 0.023 | 1000 | -0.031 to 0.081 |
| Clinical + imaging model | 0.631 | 0.612 | 0.019 | 1000 | -0.039 to 0.075 |
| Clinical + electrophysiological model | 0.739 | 0.723 | 0.015 | 1000 | -0.039 to 0.070 |
| Combined model | 0.75 | 0.731 | 0.018 | 1000 | -0.034 to 0.072 |


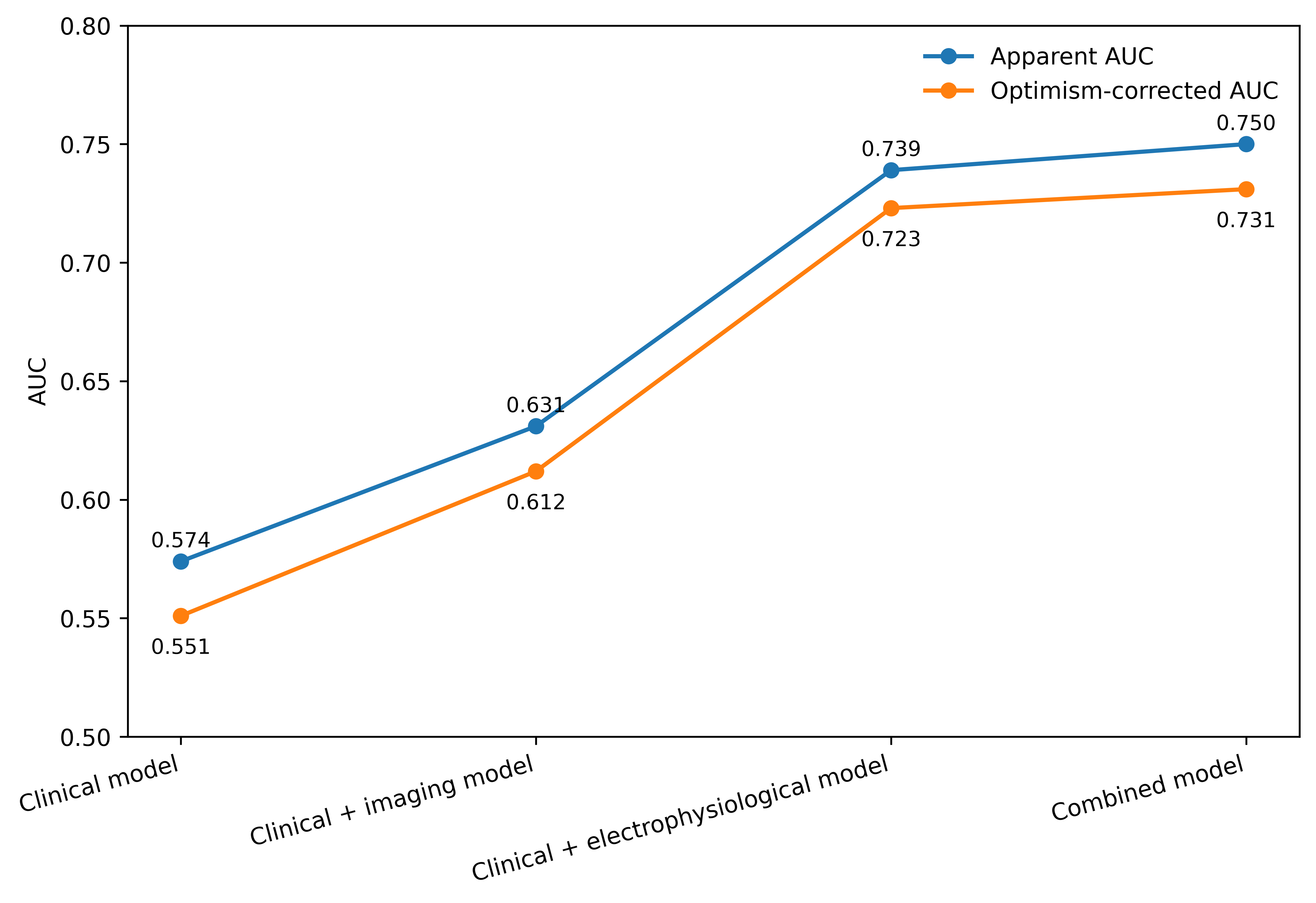


**Supplementary Figure S1. Apparent and optimism-corrected AUC values across the four prediction models.**

The figure compares the apparent area under the receiver operating characteristic curve (AUC) and the optimism-corrected AUC for the clinical model, clinical + imaging model, clinical + electrophysiological model, and combined model. The relatively small differences between apparent and optimism-corrected AUC values indicate limited overoptimism and support reasonable internal stability of the models.


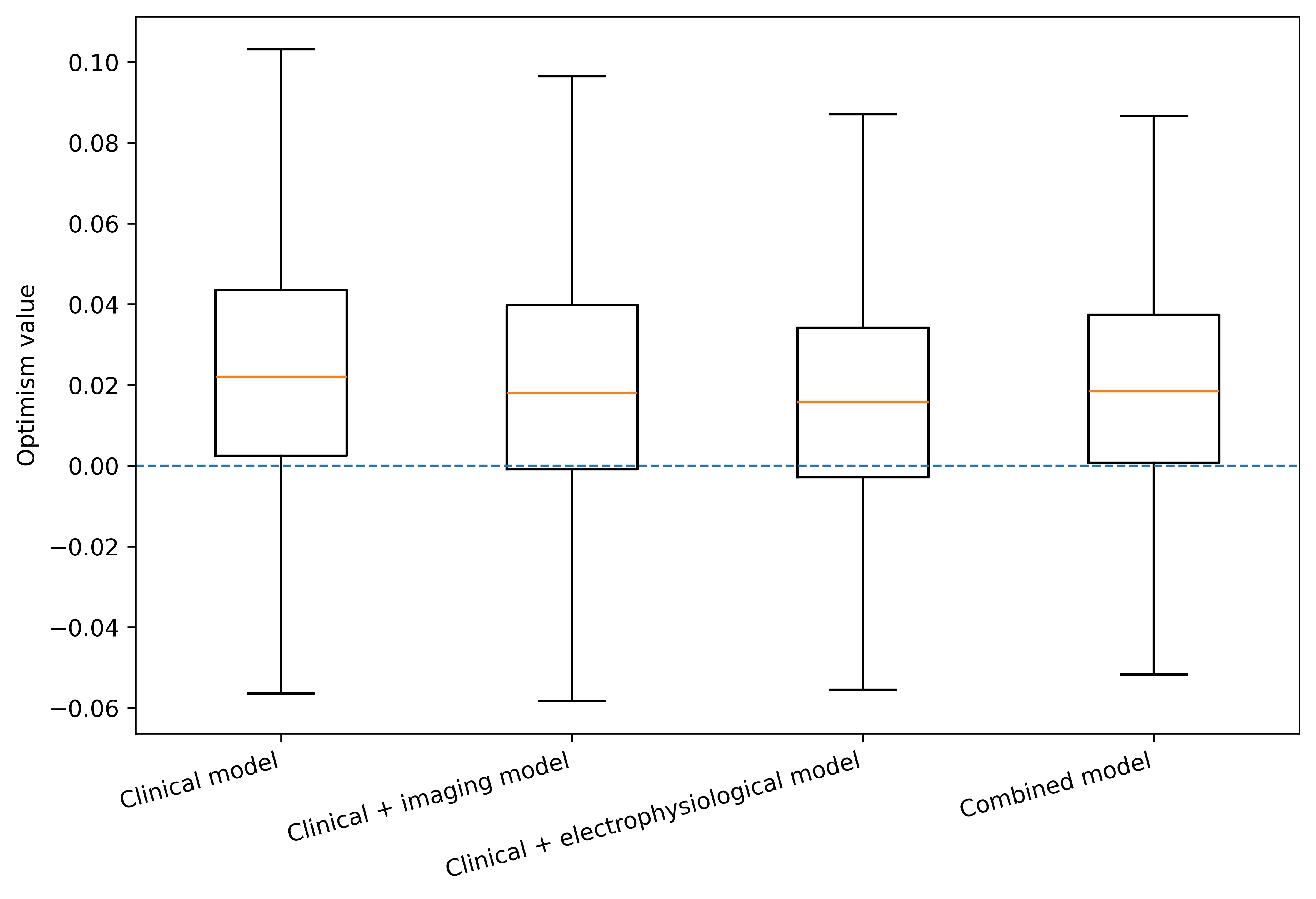


**Supplementary Figure S2. Distribution of optimism values across bootstrap resamples for the four prediction models.**

Boxplots show the distribution of optimism values derived from bootstrap resampling for the clinical model, clinical + imaging model, clinical + electrophysiological model, and combined model. The generally small optimism values across models suggest limited overfitting and support the robustness of internal model performance.
